# Local Generalized (*α,β*)-Derivations

**DOI:** 10.1155/2014/805780

**Published:** 2014-02-16

**Authors:** Ajda Fošner

**Affiliations:** Faculty of Management, University of Primorska, Cankarjeva 5, SI-6104 Koper, Slovenia

## Abstract

We study local generalized (*α,β*)-derivations on algebras generated by their idempotents and give some important applications of our results.

## 1. Introduction

A linear mapping *d* on an algebra *𝒜* is called a local derivation if for every *a* ∈ *𝒜* there exists a derivation *d*_*a*_ : *𝒜* → *𝒜* depending on *a* such that *d*(*a*) = *d*_*a*_(*a*). This notion was introduced independently by Kadison [1] and Larson and Sourour [2]. In [1] Kadison investigated continuous local derivations on von Neumann algebras. He proved that if *𝒜* is a von Neumann algebra and *ℳ* a dual *𝒜*-module, then all norm-continuous local derivations from *𝒜* into *ℳ* are derivations. This research was motivated by problems concerning the Hochschild cohomology of operator algebras. On the other hand, Larson and Sourour [2] proved that every local derivation on the algebra of all bounded linear operators on a Banach space is a derivation.

Of course, every derivation on an algebra *𝒜* is a local derivation. But the converse is in general not true. Kadison [1] constructed an example (due to C. Jensen) of an algebra (not an operator algebra) which has nontrivial local derivations. Moreover, there are examples of local derivations on operator algebras in the literature, for example, using the subalgebra of 3 × 3 complex matrices consisting of constant multiples of the identity plus strictly upper triangular matrices.

In the last few decades a lot of work has been done on local mappings on some algebras. The results show that, in many important cases, local mappings of some certain class of transformations on a given algebra are global (see [3]). In the context of derivations, the relation between local derivations and derivations was widely studied by several authors (see, e.g., [1, 2, 4–10] and the references therein). Recently, Hadwin and Li proved that every local derivation from an algebra *𝒜* which is generated by its idempotents into any *𝒜*-bimodule is a derivation. This motivated us to study local generalized (*α*, *β*)-derivations on algebras generated by their idempotents.

Before continuing, let us fix the notation and write some basic definitions which we will need in our further investigation. Throughout the paper, *𝒜* will be an algebra and *ℳ* will be an *𝒜*-bimodule. A linear mapping *d* : *𝒜* → *ℳ* is called a derivation if

(1) $$\begin{array}{c} d\left(ab\right)=d\left(a\right)b+ad\left(b\right),\;a,b\in 𝒜. \end{array}$$

In [11], Brešar defined the notion of generalized derivation as follows. A linear mapping *g* : *𝒜* → *ℳ* is a generalized derivation if there exists a derivation *d* : *𝒜* → *ℳ* such that

(2) $$\begin{array}{c} g\left(ab\right)=g\left(a\right)b+ad\left(b\right),\;a,b\in 𝒜. \end{array}$$

In this case we say that *g* is a generalized derivation associated with a derivation *d* (or simply, *g* is a generalized *d*-derivation). On the other hand, Nakajima [12] defined generalized derivations without using the corresponding derivations as follows. Let *g* : *𝒜* → *ℳ* be a linear mapping and *m* an element of *ℳ*. A pair (*g*; *m*) is called a generalized derivation if

(3) $$\begin{array}{c} g\left(ab\right)=g\left(a\right)b+ag\left(b\right)+amb,\;a,b\in 𝒜. \end{array}$$

In particular, if *𝒜* has a unit 1 ∈ *𝒜*, then *m* = −*g*(1) and *g* is a generalized derivation if it satisfies

(4) $$\begin{array}{c} g\left(ab\right)=g\left(a\right)b+ag\left(b\right)-ag\left(1\right)b,\;a,b\in 𝒜. \end{array}$$

Since we will deal just with unital algebras, we will say that a linear mapping *g* : *𝒜* → *ℳ* is a generalized derivation if and only if it satisfies the above condition. We refer the readers to [12], where they can find more information about generalized derivations.

Let *α*, *β* be endomorphisms of a unital algebra *𝒜* and *I*_*𝒜*_ the identity map on *𝒜*. Motivated by the above notions, we define (*α*, *β*)-derivations as linear mappings *d* : *𝒜* → *ℳ* satisfying

(5) $$\begin{array}{c} d\left(ab\right)=d\left(a\right)\alpha \left(b\right)+\beta \left(a\right)d\left(b\right),\;a,b\in 𝒜. \end{array}$$

Further, a linear mapping *g* : *𝒜* → *ℳ* is called a generalized (*α*, *β*)-derivation if

(6) g(ab)=g(a)α(b)+β(a)g(b)−β(a)g(1)α(b),a,b∈𝒜.

Of course, (*I*_*𝒜*_, *I*_*𝒜*_)-derivation (generalized (*I*_*𝒜*_, *I*_*𝒜*_)-derivation, resp.) is just a derivation (generalized derivation, resp.). The next example will show that there exist (*α*, *β*)-derivations which are not derivations.

**Example 1**

Let *𝒜* be an algebra with a nontrivial central idempotent *e*. Let us define *d*(*a*) = *ea*, *α*(*a*) = *a* − *ea* for all *a* ∈ *𝒜*, and *β* = *I*_*𝒜*_. Then *d* is an (*α*, *β*)-derivation which is not a derivation since *d*(*ee*) = *ee* *e* ≠ 2*ee* *e* = (*ee*)*e* + *e*(*ee*) = *d*(*e*)*e*  +  *ed*(*e*). Moreover, if *𝒜* is a semiprime algebra and *α* ≠ *I*_*𝒜*_ is an endomorphism of *𝒜*, then *d* = *I*_*𝒜*_ − *α* is an (*α*, *I*_*𝒜*_)-derivation but not a derivation.

The definition of local generalized (*α*, *β*)-derivations (local (*α*, *β*)-derivations, resp.) can be self-explanatory, A linear mapping *g* : *𝒜* → *ℳ* is called a local generalized (*α*, *β*)-derivation (local (*α*, *β*)-derivation, resp.) if for every *a* ∈ *𝒜* there exists a generalized (*α*, *β*)-derivation ((*α*, *β*)-derivation, resp.) *g*_*a*_ : *𝒜* → *ℳ* depending on *a* such that *g*(*a*) = *g*_*a*_(*a*).

In the following, we assume that all algebras are unital topological algebras and all bimodules are unital topological bimodules. Recall that a topological algebra is an associative algebra equipped with a vector space topology compatible with its ring structure, in the sense that the ring multiplication is separately continuous. Let *ℳ* be an *𝒜*-bimodule. If *ℳ* is a topological vector space and *𝒜* a topological algebra such that the module multiplications are separately continuous, then we say that *ℳ* is a topological *𝒜*-bimodule.

## 2. Local Generalized (*α*, *β*)-Derivations

Let *ℳ* be an *𝒜*-bimodule and *ℐ* an ideal of *𝒜*. We say that *ℐ* is a separating set of *ℳ* if, for all *m*, *n* ∈ *ℳ*, *mℐ* = {0} implies *m* = 0 and *ℐn* = {0} implies *n* = 0. In the following, *α*, *β* : *𝒜* → *𝒜* will be unital homomorphisms. For an idempotent *e* ∈ *𝒜*, we write *e* ^⊥^ = 1 − *e*.

**Lemma 2**

Let *g* be a linear mapping from an algebra *𝒜* into an *𝒜*-bimodule *ℳ*. Then for every *a* ∈ *𝒜* and all idempotents *e*, *f* ∈ *𝒜*, the following are equivalent:

- (1 − *β*(*e*))*g*(*ea* *f*)(1 − *α*(*f*)) = 0,
- *g*(*ea* *f*) = *g*(*ea*)*α*(*f*) + *β*(*e*)*g*(*af*) − *β*(*e*)*g*(*a*)*α*(*f*).

**Proof**

Obviously, (ii) implies (i). So, assume that (i) holds for every *a* ∈ *𝒜* and all idempotents *e*, *f* ∈ *𝒜*. Then

(7) $$\begin{array}{c} \beta \left(e\right)g\left(eaf\right)\alpha {\left(f\right)}^{⊥}=g\left(eaf\right)\alpha {\left(f\right)}^{⊥}. \end{array}$$

On the other hand,

(8) β(e)g(eaf)α(f)⊥=β(e)g((1−e⊥)af)α(f)⊥=β(e)(g(af)−g(e⊥af))α(f)⊥=β(e)g(af)α(f)⊥.

This yields that

(9) $$\begin{array}{c} \beta \left(e\right)g\left(af\right)\alpha {\left(f\right)}^{⊥}=g\left(eaf\right)\alpha {\left(f\right)}^{⊥} \end{array}$$

for every *a* ∈ *𝒜* and all idempotents *e*, *f* ∈ *𝒜*. Therefore,

(10) g(eaf)−β(e)g(af)  =(g(eaf)−β(e)g(af))(α(f)+α(f)⊥)  =(g(eaf)−β(e)g(af))α(f)  =(g(ea(1−f⊥))−β(e)g(af))α(f)  =g(ea)α(f)−β(e)g(af⊥)α(f)−β(e)g(af)α(f)  =g(ea)α(f)−β(e)g(a)α(f)

for every *a* ∈ *𝒜* and all idempotents *e*, *f* ∈ *𝒜*.

**Lemma 3**

If *g* is a linear mapping from an algebra *𝒜* into an *𝒜*-bimodule *ℳ* such that *g*(*ea* *f*) = *g*(*ea*)*α*(*f*) + *β*(*e*)*g*(*af*) − *β*(*e*)*g*(*a*)*α*(*f*) for every *a* ∈ *𝒜* and all idempotents *e*, *f* ∈ *𝒜*, then

(11) g(e1⋯emaf1⋯fn)=g(e1⋯ema)α(f1⋯fn)+β(e1⋯em)g(af1⋯fn)−β(e1⋯em)g(a)α(f1⋯fn)

for every *a* ∈ *𝒜* and all idempotents *e*_1_,…, *e*_*m*_, *f*_1_,…, *f*_*n*_ ∈ *𝒜*.

**Proof**

Using the induction on *n*, we first prove that for every *a* ∈ *𝒜* and all idempotents *e*, *f*_1_,…, *f*_*n*_ ∈ *𝒜*,

(12) g(eaf1⋯fn)=g(ea)α(f1⋯fn)+β(e)g(af1⋯fn)−β(e)g(a)α(f1⋯fn).

The case *n* = 1 is clear. Assume now that (12) holds true for *n* ≥ 1. Then

(13) g(eaf1⋯fnfn+1)  =g(eaf1⋯fn)α(fn+1)+β(e)g(af1⋯fnfn+1)   −β(e)g(af1⋯fn)α(fn+1)  =g(ea)α(f1⋯fnfn+1)+β(e)g(af1⋯fn)α(fn+1)   −β(e)g(a)α(f1⋯fnfn+1)+β(e)g(af1⋯fnfn+1)   −β(e)g(af1⋯fn)α(fn+1)  =g(ea)α(f1⋯fnfn+1)+β(e)g(af1⋯fnfn+1)   −β(e)g(a)α(f1⋯fnfn+1).

To show our lemma, we use the induction on *m*. We have just proved the case *m* = 1. So, assume that our assertion holds true for *m* ≥ 1. Then for every *a* ∈ *𝒜* and all idempotents *e*_1_,…, *e*_*m*_, *e*_*m*+1_, *f*_1_,…, *f*_*n*_ ∈ *𝒜*,

(14) g(e1e2⋯em+1af1⋯fn)  =g(e1e2⋯em+1a)α(f1⋯fn)   +β(e1)g(e2⋯em+1af1⋯fn)   −β(e1)g(e2⋯em+1a)α(f1⋯fn)  =g(e1e2⋯em+1a)α(f1⋯fn)   +β(e1)g(e2⋯em+1a)α(f1⋯fn)   +β(e1e2⋯em+1)g(af1⋯fn)   −β(e1e2⋯em)g(a)α(f1⋯fn)   −β(e1)g(e2⋯em+1a)α(f1⋯fn)  =g(e1e2⋯em+1a)α(f1⋯fn)   +β(e1e2⋯em+1)g(af1⋯fn)   −β(e1e2⋯em)g(a)α(f1⋯fn).

Our first theorem is a generalization of Theorem  2.7 in [8] (see also [7, Theorem 22]).

**Theorem 4**

Let *ℐ* be a separating set of an *𝒜*-bimodule *ℳ* contained in the algebra generated by all idempotents in *𝒜*, *α*, *β* endomorphisms of *𝒜* such that *α*(*ℐ*) = *ℐ*, *β*(*ℐ*) = *ℐ*, and let *g* : *𝒜* → *ℳ* be a linear mapping. If for every *a* ∈ *𝒜* and all idempotents *e*, *f* ∈ *𝒜*,

(15) $$\begin{array}{c} g\left(eaf\right)=g\left(ea\right)\alpha \left(f\right)+\beta \left(e\right)g\left(af\right)-\beta \left(e\right)g\left(a\right)\alpha \left(f\right), \end{array}$$

then *g* is a generalized (*α*, *β*)-derivation. In particular, if *g*(1) = 0, then *g* is an (*α*, *β*)-derivation.

The idea of the proof comes from [8, Proof of Theorem 2.7]. For the sake of completeness, we write the main steps.

**Proof**

Let *p*, *q* ∈ *ℐ* be arbitrary elements. Then, according to (15) and Lemma 3, we have

(16) $$\begin{array}{c} g\left(pq\right)=g\left(p\right)\alpha \left(q\right)+\beta \left(p\right)g\left(q\right)-\beta \left(p\right)g\left(1\right)\alpha \left(q\right). \end{array}$$

This yields that

(17) g(paq)=g((pa)q)=g(pa)α(q)+β(pa)g(q)−β(pa)g(1)α(q)

for all *a* ∈ *𝒜* since *ℐ* is an ideal of *𝒜*. On the other hand, by (15), we have

(18) $$\begin{array}{c} g\left(paq\right)=g\left(pa\right)\alpha \left(q\right)+\beta \left(p\right)g\left(aq\right)-\beta \left(p\right)g\left(a\right)\alpha \left(q\right) \end{array}$$

and, consequently,

(19) β(p)g(aq)  =β(p)g(a)α(q)+β(pa)g(q)−β(pa)g(1)α(q)  =β(p)(g(a)α(q)+β(a)g(q)−β(a)g(1)α(q)).  

Since *ℐ* is a separating set of *ℳ* and *β*(*ℐ*) = *ℐ*, it follows that

(20) $$\begin{array}{c} g\left(aq\right)=g\left(a\right)\alpha \left(q\right)+\beta \left(a\right)g\left(q\right)-\beta \left(a\right)g\left(1\right)\alpha \left(q\right) \end{array}$$

for all *a* ∈ *𝒜*. Furthermore, if *a*, *b* ∈ *𝒜* and *q* ∈ *ℐ*, then

(21) g(a(bq))  =g(a)α(bq)+β(a)g(bq)−β(a)g(1)α(bq)  =g(a)α(bq)+β(a)g(b)α(q)+β(ab)g(q)   −β(ab)g(1)α(q)−β(a)g(1)α(bq).

On the other hand,

(22) g((ab)q)=g(ab)α(q)+β(ab)g(q)−β(ab)g(1)α(q).

Therefore,

(23) g(ab)α(q)  =g(a)α(bq)+β(a)g(b)α(q)−β(a)g(1)α(bq)  =(g(a)α(b)+β(a)g(b)−β(a)g(1)α(b))α(q)

and, as above,

(24) $$\begin{array}{c} g\left(ab\right)=g\left(a\right)\alpha \left(b\right)+\beta \left(a\right)g\left(b\right)-\beta \left(a\right)g\left(1\right)\alpha \left(b\right) \end{array}$$

for all *a*, *b* ∈ *𝒜*. If *g*(1) = 0, then *g* is, obviously, an (*α*, *β*)-derivation.

Let *g* be a local generalized (*α*, *β*)-derivation from an algebra *𝒜* into an *𝒜*-bimodule *ℳ*. Suppose that *a* ∈ *𝒜* is an arbitrary element and suppose that *e*, *f* ∈ *𝒜* are idempotents. Then there exists a generalized (*α*, *β*)-derivation *g*_*eaf*_ : *𝒜* → *ℳ* such that *g*(*ea* *f*) = *g*_*eaf*_(*ea* *f*). It is also easy to see that

(25) geaf(eaf)=geaf(e)α(af)+β(e)geaf(a)α(f)+β(ea)geaf(f)−β(e)geaf(1)α(af)−β(ea)geaf(1)α(f).

Hence,

(26) (1−β(e))g(eaf)(1−α(f))  =(1−β(e))geaf(eaf)(1−α(f))=0

for every *a* ∈ *𝒜* and all idempotents *e*, *f* ∈ *𝒜*. Thus, by Lemma 2, *g* satisfies the condition (15), and, using Theorem 4, we have the next result.

**Theorem 5**

Let *ℐ* be a separating set of an *𝒜*-bimodule *ℳ* contained in the algebra generated by all idempotents in *𝒜* and let *α*, *β* be endomorphisms of *𝒜* such that *α*(*ℐ*) = *ℐ*, *β*(*ℐ*) = *ℐ*. Then every local generalized (*α*, *β*)-derivation (local (*α*, *β*)-derivation, resp.) from an algebra *𝒜* into an *𝒜*-bimodule *ℳ* is a generalized (*α*, *β*)-derivation ((*α*, *β*)-derivation, resp.).

Taking *α*, *β* = *I*_*𝒜*_, we have the next direct consequence of Theorem 5.

**Corollary 6**

Let *ℐ* be a separating set of an *𝒜*-bimodule *ℳ* contained in the algebra generated by all idempotents in *𝒜*. Then every local generalized derivation (local derivation, resp.) from an algebra *𝒜* into an *𝒜*-bimodule *ℳ* is a generalized derivation (derivation, resp.).

At the end, if *α* = *I*_*𝒜*_, then we have the next result for local generalized skew derivations, that is; linear mappings *g* : *𝒜* → *ℳ* with the property

(27) $$\begin{array}{c} g\left(ab\right)=g\left(a\right)b+\beta \left(a\right)d\left(b\right)-\beta \left(a\right)g\left(1\right)b,\;a,b\in 𝒜. \end{array}$$

**Corollary 7**

Let *ℐ* be a separating set of an *𝒜*-bimodule *ℳ* contained in the algebra generated by all idempotents in *𝒜* and let *β* be an endomorphism of *𝒜* such that *β*(*ℐ*) = *ℐ*. Then every local generalized skew derivation (local skew derivation, resp.) from an algebra *𝒜* into an *𝒜*-bimodule *ℳ* is a generalized skew derivation (skew derivation, resp.).

## 3. Applications

Our results in Section 2 hold for unital algebras that can be generated (as algebras) by their idempotents. This class of algebras contains many important algebras. For example, if *𝒜* is a unital algebra and *n* ≥ 2, a positive integer, then *M*_*n*_(*𝒜*), that is, the algebra of all *n* × *n* matrices over *𝒜*, belongs to this class (see [8, 13]). Thus, we have the next result.

**Corollary 8**

Let *α*, *β* be automorphisms of *M*_*n*_(*𝒜*). Then every local generalized (*α*, *β*)-derivation (local (*α*, *β*)-derivation, resp.) from an algebra *M*_*n*_(*𝒜*) into any *M*_*n*_(*𝒜*)-bimodule is a generalized (*α*, *β*)-derivation ((*α*, *β*)-derivation, resp.).

The next result involves local matrix algebras: an algebra *ℬ* is called a local matrix algebra if any finite subset of *ℬ* can be embedded in a subalgebra which is a matrix algebra *M*_*n*_(*𝒜*), *n* ≥ 2.

**Corollary 9**

Let *α*, *β* be automorphisms of *𝒜*. If, for any *a*_1_, *a*_2_ ∈ *𝒜*, there exists a unital subalgebra *ℬ* of *𝒜* which contains *a*_1_, *a*_2_ and is isomorphic to a matrix algebra, then every local generalized (*α*, *β*)-derivation (local (*α*, *β*)-derivation, resp.) from an algebra *𝒜* into any *𝒜*-bimodule is a generalized (*α*, *β*)-derivation ((*α*, *β*)-derivation, resp.).

Let *X* and *Y* be complex Hausdorff topological linear spaces and let *ℬ*(*X*, *Y*) be the algebra of all continuous linear mappings from *X* into *Y*. We say that a subset *𝒮* of *ℬ*(*X*, *Y*) is reflexive if *T* ∈ *𝒮* whenever *T* ∈ *ℬ*(*X*, *Y*) and $Tx\in \bar{𝒮x}$ for any *x* ∈ *X*, where $\bar{𝒮x}$ denotes the topological closure of *𝒮x*. By a subspace lattice on *X* we mean a collection *ℒ* of closed subspaces of *X* containing {0} and *X* such that, for each family {*L*_*ϵ*_} of elements of *ℒ*, both ⋂*L*_*ϵ*_ and ⋁*L*_*ϵ*_ belong to *ℒ*, where ⋁ denotes the closed linear span of {*L*_*ϵ*_}. If *ℒ* is a subspace lattice, then we denote the algebra of all operators on *X*, that leave invariant each element of *ℒ* by alg*ℒ*. A totally ordered subspace lattice *𝒩* is called a nest and the associated reflexive algebra alg*𝒩* is called a nest algebra.

Now we consider local generalized (*α*, *β*)-derivations on a reflexive subalgebra in a factor von Neumann algebra. The proof of the following corollaries uses Theorem 4 and arguments similar to those in the proof of [8, Theorem 2.17, Theorem 2.18].

**Corollary 10**

Let *ℒ* be a subspace lattice in a factor von Neumann algebra *ℳ* on *H* with ⋂{*L* ∈ *ℒ* : 0 ⊂ *L*} ≠ 0 and ⋁{*L* ∈ *ℒ* : *L* ⊂ *H*} ≠ *H* and let *α*, *β* be automorphisms of *ℳ*∩*algℒ*. Then every local generalized (*α*, *β*)-derivation (local (*α*, *β*)-derivation, resp.) from *ℳ*∩*algℒ* into *ℳ* is a generalized (*α*, *β*)-derivation ((*α*, *β*)-derivation, resp.).

**Corollary 11**

Let *𝒩* be a nest in a factor von Neumann algebra *ℳ* on *H* and let *α*, *β* be automorphisms of *ℳ*∩*alg𝒩*. Then every local generalized (*α*, *β*)-derivation (local (*α*, *β*)-derivation, resp.) from *ℳ*∩*alg𝒩* into *ℳ* is a generalized (*α*, *β*)-derivation ((*α*, *β*)-derivation, resp.).

Suppose that *𝒜* is topologically generated by its idempotents (i.e., the subalgebra of *𝒜* generated by its idempotents is dense in *𝒜*) and suppose that *g* : *𝒜* → *ℳ* is a continuous local generalized (*α*, *β*)-derivation, where *α*, *β* are automorphisms of *𝒜*. Let *a* = ∑_*i*=1_^*m*^ *λ*_*i*_∏_*j*=1_^*t*_*i*_^ *e*_*j*_^(*i*)^, *b* = ∑_*k*=1_^*n*^ *μ*_*k*_∏_*l*=1_^*s*_*k*_^ *f*_*l*_^(*k*)^ for some idempotents *e*_*j*_^(*i*)^, *f*_*l*_^(*k*)^ ∈ *𝒜* and some scalars *λ*_*i*_, *μ*_*k*_. Then, by Lemma 3, we have *g*(*ab*) = *g*(*a*)*α*(*b*) + *β*(*a*)*g*(*b*) − *α*(*a*)*g*(1)*β*(*b*) and since *g* is continuous and *𝒜* is generated by its idempotents, we have the following proposition.

**Proposition 12**

Let *α*, *β* be automorphisms of *𝒜*. If *𝒜* is topologically generated by its idempotents and *ℳ* is a topological *𝒜*-bimodule, then every continuous local generalized (*α*, *β*)-derivation (local (*α*, *β*)-derivation, resp.) from *𝒜* into *ℳ* is a generalized (*α*, *β*)-derivation ((*α*, *β*)-derivation, resp.).

Hadwin and Li proved that if *𝒩* is a nest in a von Neumann algebra *ℳ* and *𝒜* = *ℳ*∩alg*𝒩*, then the linear span of all idempotents in *𝒜* is w*-dense in *𝒜* (see [8, Proposition 2.3]). Thus, by Proposition 12, we have the next corollary.

**Corollary 13**

Let *𝒩* be a nest in a von Neumann algebra *ℳ*, *𝒜* = *ℳ*∩alg*𝒩*, and let *α*, *β* be automorphisms of *𝒜*. Then every w*-continuous local generalized (*α*, *β*)-derivation (resp. local (*α*, *β*)-derivation) from *𝒜* into *ℳ* is a generalized (*α*, *β*)-derivation (resp. (*α*, *β*)-derivation).

At the end, let us prove that the set of all continuous generalized (*α*, *β*)-derivations from *𝒜* into *ℳ*, denoted by *GDer*_*α*,*β*_(*𝒜*, *ℳ*), is reflexive.

**Corollary 14**

Let *𝒜*, *ℳ*, *α*, *β* be as in Theorem 4. Then *GDer*_*α*,*β*_(*𝒜*, *ℳ*) is reflexive.

**Proof**

Let *g* : *𝒜* → *ℳ* be a continuous linear mapping such that for any *x* ∈ *𝒜*,

(28) $$\begin{array}{c} g\left(x\right)\in \bar{GDer_{\alpha ,\beta }\left(𝒜,ℳ\right)x}. \end{array}$$

Now, let *e*, *f* ∈ *𝒜* be arbitrary idempotents, *a* any element from *𝒜*, and *x* = *ea* *f*. Then, according to above observations, there exists a sequence {*g*_*n*_}_*n*=1_^*∞*^ ⊂ *GDer*_*α*,*β*_(*𝒜*, *ℳ*) such that

(29) $$\begin{array}{c} \underset{n\to \infty }{\lim}g_{n}\left(x\right)=g\left(x\right). \end{array}$$

Therefore,

(30) (1−β(e))g(eaf)(1−α(f))  =(1−β(e))lim⁡n→∞gn(eaf)(1−α(f))=0.

According to Lemma 2 and Theorem 4, this yields that *g* is a generalized (*α*, *β*)-derivation; that is, *g* ∈ *GDer*_*α*,*β*_(*𝒜*, *ℳ*). The proof is completed.
